# Differential impacts of DNA repair machinery on fluoroquinolone persisters with different chromosome abundances

**DOI:** 10.1128/mbio.00374-24

**Published:** 2024-04-02

**Authors:** Juechun Tang, Allison M. Herzfeld, Gabrielle Leon, Mark P. Brynildsen

**Affiliations:** 1 Department of Chemical and Biological Engineering, Princeton University, Princeton, New Jersey, USA; 2 Department of Molecular Biology, Princeton University, Princeton, New Jersey, USA; 3 Rutgers Robert Wood Johnson Medical School, Piscataway, New Jersey, USA; Racah Institute of Physics and the Harvey, The Hebrew University of Jerusalem, Jerusalem, Israel

**Keywords:** persistence, levofloxacin, ciprofloxacin, *xseA*, *xseB*, *uvrD*, *recN*, *lexA*

## Abstract

DNA repair machinery has been found to be indispensable for fluoroquinolone (FQ) persistence of *Escherichia coli*. Previously, we found that cells harboring two copies of the chromosome (2Chr) in stationary-phase cultures were more likely to yield FQ persisters than those with one copy of the chromosome (1Chr). Furthermore, we found that RecA and RecB were required to observe that difference, and that loss of either more significantly impacted 2Chr persisters than 1Chr persisters. To better understand the survival mechanisms of persisters with different chromosome abundances, we examined their dependencies on different DNA repair proteins. Here, we show that *lexA3* and ∆*recN* negatively impact the abundances of 2Chr persisters to FQs, without significant impacts on 1Chr persisters. In comparison, ∆*xseA*, ∆*xseB*, and ∆*uvrD* preferentially depress 1Chr persistence to levels that were near the limit of detection. Collectively, these data show that the DNA repair mechanisms used by persisters vary based on chromosome number, and suggest that efforts to eradicate FQ persisters will likely have to take heterogeneity in single-cell chromosome abundance into consideration.

**IMPORTANCE:**

Persisters are rare phenotypic variants in isogenic populations that survive antibiotic treatments that kill the other cells present. Evidence has accumulated that supports a role for persisters in chronic and recurrent infections. Here, we explore how an under-appreciated phenotypic variable, chromosome copy number (#Chr), influences the DNA repair systems persisters use to survive fluoroquinolone treatments. We found that #Chr significantly biases the DNA repair systems used by persisters, which suggests that #Chr heterogeneity should be considered when devising strategies to eradicate these troublesome bacterial variants.

## INTRODUCTION

Bacterial persisters, which are subpopulations of isogenic bacterial cultures that can tolerate lethal doses of antibiotics when the majority of cells cannot, constitute a major threat to public health due to their abilities to repopulate infection sites after the conclusion of treatments (1–6). Indeed, multiple lines of clinical evidence have indicated the role of bacterial persisters in recalcitrant, hard-to-treat infections (5, 7–10). For instance, 19% of *Pseudomonas aeruginosa* isolates from cystic fibrosis (CF) patients were classified as high-persistence variants, and patients with those variants were treated with antibiotics more frequently than those without such variants (9). Similarly, high-persistence variants were observed in clinical isolates of uropathogenic *Escherichia coli* obtained from patients with urinary tract infections (UTIs) (7, 11). In addition, evidence has emerged suggesting that recurrent UTIs are typically caused by the original infecting strain, rather than another strain or resistant mutant (12). Furthermore, persistence is suspected to underlie the latency of *Mycobacterium tuberculosis* in tuberculosis patients (8, 10, 13), the recurrence of non-typhoidal *Salmonella* infections (14), and many other infections caused by bacterial pathogens, such as *Streptococcus pneumoniae* and *Staphylococcus aureus* (15, 16). Importantly, several groups have shown that persisters facilitate the development of resistant mutants (17–20), deepening the clinical concern of this phenotype.

Persisters have been observed in almost all bacterial pathogens (21) and their abundances can vary based on the antibiotics used (22–25). For example, Luidalepp and colleagues observed persistence to amikacin that was far lower than that of persistence to ampicillin (AMP) or norfloxacin (22). While studying persister formation from nutrient transitions, Amato and Brynildsen observed that dual treatment with AMP and ofloxacin yielded approximately 10-fold fewer persisters than either individual treatment alone, which suggested that the minority of persisters formed were multidrug tolerant (23). Dörr and colleagues identified a persister formation pathway for fluoroquinolones (FQs) that involved SOS induction of TisB that was not involved in AMP or streptomycin persistence of the same cultures (25). Furthermore, even within the same drug class, persister levels can vary, as evidenced by a recent study of FQs (24). Overall, these studies and others have established that while persisters can be tolerant to multiple antibiotics, not all persisters are multidrug tolerant.

FQs constitute a class of broad-spectrum antibiotics that are widely used for the treatment of respiratory and urinary tract infections (26). Further, FQs appear on the World Health Organization’s model list of essential medicines, including ciprofloxacin (CIP), levofloxacin (LEVO), and moxifloxacin (27). Importantly, while many other classes of antibiotics, such as β-lactams and aminoglycosides, are generally effective in killing only actively growing bacteria (28–30), FQs can kill both growing and growth-inhibited bacteria (31), even though their efficacy is better against growing bacteria (31–34). FQs kill by trapping type II topoisomerases in cleaved complexes with DNA, which can lead to DNA damage (35–39). Recently, we have shown that the extent to which DNA gyrase is stabilized by FQs in cleaved complexes across the chromosome is highly predictive of persister levels in stationary-phase *E. coli* populations (24). Furthermore, it has been observed that FQ persisters are not exempt from DNA damage in either growing or non-growing populations (19, 33, 40), and numerous DNA repair genes have been identified as important to FQ persister survival (19, 20, 41, 42). Additionally, we discovered that chromosome copy number (#Chr) is a phenotypic trait that influences FQ persistence, because it dictates whether bacteria have a homologous chromosome to serve as a template for homologous recombination (HR) (41). Specifically, we showed that bacteria with two copies of the chromosome (2Chr) were more likely to be persisters than bacteria with one copy of the chromosome (1Chr) in stationary-phase *E. coli* cultures, and that *recA* and *recB* were required to observe those differences (41). Interestingly, 1Chr and 2Chr persister levels were both impacted by Δ*recA* and Δ*recB*; however, 2Chr persistence depended on *recA* and *recB* to a greater extent (~30- and ~100-fold declines in 1Chr persisters compared with ~300- and ~1,000-fold declines in 2Chr persisters in *∆recA* and *∆recB*, respectively). These data suggested that the survival mechanisms of individual FQ persisters varied, and that those mechanisms were impacted by heterogeneity in single-cell chromosome abundance (e.g., 1Chr vs 2Chr).

Here, we focused on examining the dependencies of 1Chr and 2Chr FQ persisters on DNA repair systems previously identified to impact FQ persistence. *recA*, *recB*, *recN*, *lexA*, *uvrD*, *xseA*, and *xseB* were genes identified to be important to FQ persistence in stationary-phase cultures (19, 20, 25, 32, 41–45). RecA binds to single-stranded DNA (ssDNA) that occurs from the processing of DNA breaks, which then allows it to perform its two canonical functions, identifying homologous regions of DNA for recombinatorial repair and promoting the self-cleavage of LexA to initiate the SOS response (46, 47). RecB forms a complex with RecC and RecD (RecBCD) that is known as exonuclease V (ExoV), which contains helicase and exonuclease activities (48, 49). RecBCD binds to DNA double-strand breaks (DSBs) and proceeds to unwind and degrade DNA until it encounters a Chi site, where asymmetric nuclease activity produces the ssDNA tail to which RecBCD promotes RecA binding (48, 49). RecN, like RecA and RecB, is a member of the SOS regulon, and it is critical for the repair of DSBs (50). Specifically, RecN serves as a cohesive factor that maintains contacts between sister chromosomes during HR (51). LexA is the transcriptional repressor of the SOS regulon that degrades itself with the assistance of ssDNA-bound RecA to express a variety of DNA repair genes (52). UvrD is known as DNA helicase II and it is involved in a variety of DNA repair pathways that include methyl-directed mismatch repair (MMR) and nucleotide excision repair (NER) (53–57), as well as antirecombination where it helps to resolve mismatches present in RecA-mediated strand exchange (58). XseA and XseB are components of exonuclease VII (ExoVII), a single-strand exonuclease (ssExo) that was found recently to be able to remove 5´ tyrosyl adducts covalently bound to the phosphate backbone of DNA (59), which mimic the covalent bond of Tyr122 of GyrA to DNA that is stabilized by FQs (60–62). Given the knowledge base, it was not surprising that we found that Δ*recA* and Δ*recB* more highly impacted the survival of 2Chr compared to 1Chr cells treated with FQ (41); however, whether such bias with #Chr existed for the other DNA repair mutants was unknown. Here, we assessed how Δ*recN, lexA3* (an uncleavable mutant) (63), Δ*uvrD*, Δ*xseA*, and Δ*xseB* impacted FQ persistence of 1Chr and 2Chr cells. Interestingly, *recN* and *lexA3* were found to be important for 2Chr persistence, but did not appreciably impact 1Chr persistence, whereas critical roles for *uvrD*, *xseA*, and *xseB* in 1Chr persisters that exceeded their impacts in 2Chr persisters were revealed. Together, these data demonstrated that bias in DNA repair systems used by 1Chr and 2Chr FQ persisters goes far beyond RecA and RecB.

## RESULTS

### Loss of ExoVII reduces LEVO and CIP persister levels

**Fig 1:**
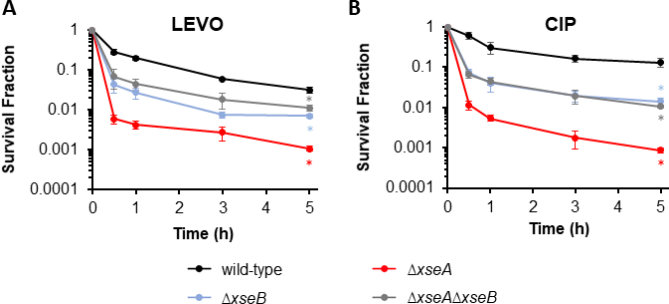
Exonuclease VII mutants show impaired persistence levels to FQ treatments. Wild type, ∆*xseA*, ∆*xseB*, or ∆*xseA*∆*xseB* were grown to stationary phase as described in Materials and Methods and treated with (**A**) 5 μg/mL LEVO or (**B**) 1 μg/mL CIP. Data denote means ± SEMs for three or more biological replicates. Statistical analyses were performed using one-way analysis of variance assessing the effects of gene deletion to log-transformed survival fraction after 5 h FQ treatment followed by Tukey honestly significant difference (HSD) *post hoc* tests for multiple comparisons [LEVO: *F*(3,18) = 94.2, *P* = 3.4e − 11; CIP: *F*(3,9) = 99.7, *P* = 5.7e − 6]. Asterisk (⁠∗) denotes statistical significance (adjusted *P* < 0.05) between indicated mutant strain and wild type.

Recently, XseA and XseB were identified from a screen for persistence to tosufloxacin where they were not assessed with LEVO or CIP, the most clinically used FQs (64), and not confirmed with genetic complementation (45). Therefore, we first sought to assess the impacts of ExoVII mutants on LEVO and CIP persistence here. ExoVII is composed of one monomer of XseA in complex with four to six monomers of XseB (41). To evaluate the contributions of ExoVII to FQ persistence, we performed assays with ∆*xseA,* ∆*xseB,* and ∆*xseA*∆*xseB* challenged with 5 µg/mL LEVO (>250× minimum inhibitory concentration (MIC) for MG1655, > 600× MIC for ExoVII mutants, Fig. S1). Following 5 h of LEVO treatment, we observed significant declines in survival in all ExoVII mutants compared to wild type (WT) (Fig. 1A). Specifically, ∆*xseA* showed a ~30-fold reduction in persister levels, whereas ∆*xseB* and ∆*xseA*∆*xseB* exhibited ~5- and 3-fold reductions, respectively. Treatment with 1 µg/mL CIP yielded similar results (Fig. 1B), whereas untreated controls showed that decreases in survival were due to FQ treatment (Fig. S2A). It is worth noting that ∆*xseB* and ∆*xseA*∆*xseB* showed higher survival compared to ∆*xseA* alone (Fig. 1). These data suggested that XseB in the absence of its binding partner XseA could be detrimental to survival following FQ treatment, whereas XseA in the absence of XseB exhibited survival similar to that of a strain devoid of both subunits. Previous studies have found that the binding of XseA to DNA requires XseB, despite the DNA-binding domain itself being located on XseA (65). Such a mechanism is consistent with Δ*xseB* and Δ*xseA*Δ*xseB* producing comparable results in persistence assays (Fig. 1), since an inability to bind its substrate would compromise its exonuclease activity. However, the specific reason as to why Δ*xseA* exhibits lower persister levels than Δ*xseA*Δ*xseB* and Δ*xseB* in the stationary-phase populations considered here remains to be determined. We note that when Δ*xseA* and Δ*xseB* were assessed for tosufloxacin persistence, the time points were acquired at 24-h intervals and a difference between the strains was not observed (45).

**Fig 2:**
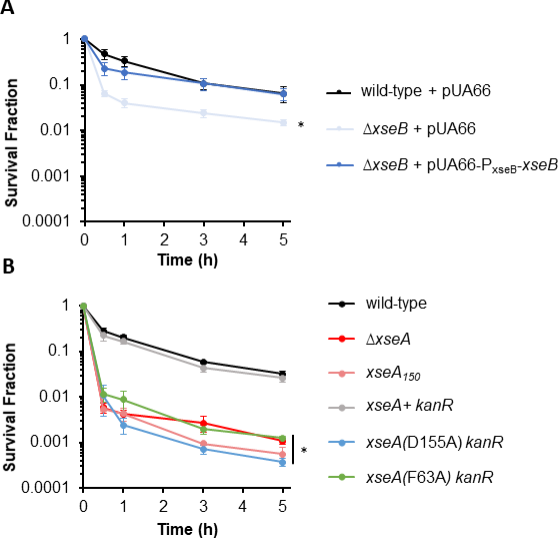
Complementation and genomic replacement establish the importance of ExoVII to FQ persistence. All strains were grown to stationary phase and treated with 5 µg/mL LEVO for 5 h. (**A**) Complementation of *xseB* via a low copy plasmid restores persistence to wild-type levels. (**B**) Replacement of *xseA* via a genomic integration technique. The nonfunctional truncated *xseA*, *xseA*_*150*_, and mutations in the DNA-binding domain [*xseA* (F63A) *kanR*] or catalytic domain [*xseA* (D155A) *kanR*] of XseA showed persister levels similar to that of ∆*xseA*, whereas restoration of the wild-type allele (*xseA*+ *kanR*) did not have an impact on persistence. Data denote means ± SEM for three or more biological replicates. Statistical analyses were performed using one-way analysis of variance at 5 h FQ treatment time point followed by Tukey HSD *post hoc* tests for multiple comparisons [XseB: *F*(2,10) = 5.8, *P* = 0.021; XseA: *F*(5,20) = 80.8, *P* = 1.4e − 12]. Asterisk (⁠∗) denotes statistical significance (adjusted *P* < 0.05) in log-transformed survival between indicated mutants (+/− plasmid) and wild type (+/− plasmid).

To confirm the role of ExoVII in FQ persistence, we performed complementation experiments. Complementation of XseB on a low copy plasmid expressed from its native promoter in Δ*xseB* restored FQ persister levels to those of wild type, whereas an empty vector could not (Fig. 2A). Plasmid-based complementation of XseA expressed from its native promoter or a strong inducible (P*_T5_*) promoter in Δ*xseA* failed to completely restore persistence to wild-type levels, which we postulated was due to reduced ExoVII activity with an overabundance of XseA with respect to XseB that was observed previously (66). In addition, overexpression of XseA was previously reported to be toxic (67), which could also explain the difficulties with plasmid-based complementation. To circumvent this technical hurdle, we generated MG1655 *xseA*_150_, a nonfunctional truncated mutant, whose FQ persister levels matched those of Δ*xseA* (Fig. 2B), and performed genomic replacement experiments with that strain (Materials and Methods). Restoration of the wild-type allele with *kanR* (kanamycin resistance gene) adjacent to it returned FQ persister levels to those of wild type, whereas integration of an allele bearing a mutation to the catalytic domain of XseA (D155A) (65) flanked by *kanR* failed to do so (Fig. 2B). Furthermore, replacement of XseA with a DNA binding mutant of XseA (F63A) (65) yielded FQ persister levels similar to Δ*xseA* and *xseA*_150_ (Fig. 2B). These data demonstrated that ExoVII was an important DNA repair system for LEVO and CIP persisters in stationary-phase cultures, which agrees with previous results with tosufloxacin (45).

### Nonredundant function of ExoVII in FQ persistence

**Fig 3:**
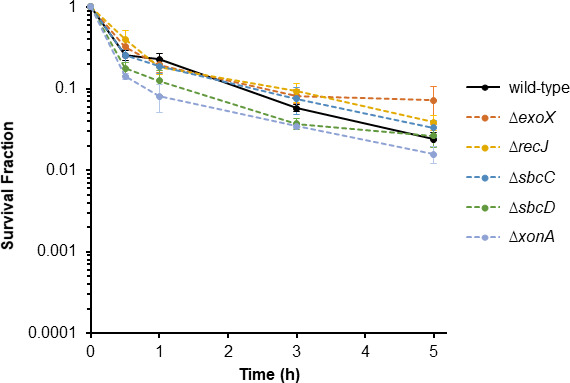
Exonucleases with redundant function with ExoVII for other processes do not impact FQ persistence. Δ*xonA*, Δ*exoX*, Δ*recJ*, Δ*sbcC*, and Δ*sbcD* were grown to stationary phase and treated with 5 µg/mL LEVO for 5 h. All tested mutants showed comparable survival as wild type. Data denote means ± SEMs for three or more biological replicates. Statistical analyses were performed using one-way analysis of variance assessing the effects of gene deletion to survival fraction at 5 h FQ treatment time point [*F*(5,15) = 1.6, *P* = 0.2].

Beyond its ability to process DNA ends with 5´ tyrosyl adducts, ExoVII has been implicated as a participant in MMR, repair of UV-induced damage, and fusion of replication forks (54, 68, 69). Notably, with these other functions, ExoVII has exhibited redundancy with a variety of other exonucleases (XonA, ExoX, RecJ, SbcCD), where removal of several is generally needed to observe impacts (68–71). To assess whether the other exonucleases known to exhibit redundancy with ExoVII also influence FQ persistence, we tested knockout mutants of *xonA*, *exoX*, *recJ, sbcC,* and *sbcD* (MICs of mutants were approximately the same as WT, Fig. S1). Results revealed that Δ*xonA*, Δ*exoX*, Δ*recJ*, Δ*sbcC*, and Δ*sbcD* had comparable persister levels as wild type (Fig. 3). Untreated controls demonstrated that FQ treatment was responsible for the observed culturability losses (Fig. S2B). Together, these data show that ExoVII is different in its importance to FQ persistence with respect to other nucleases it has exhibited redundancy with for other functions, because it is the sole nuclease whose individual deletion reduces FQ persistence levels.

### DNA repair proteins differentially impact FQ persistence of 1Chr and 2Chr cells

**Fig 4:**
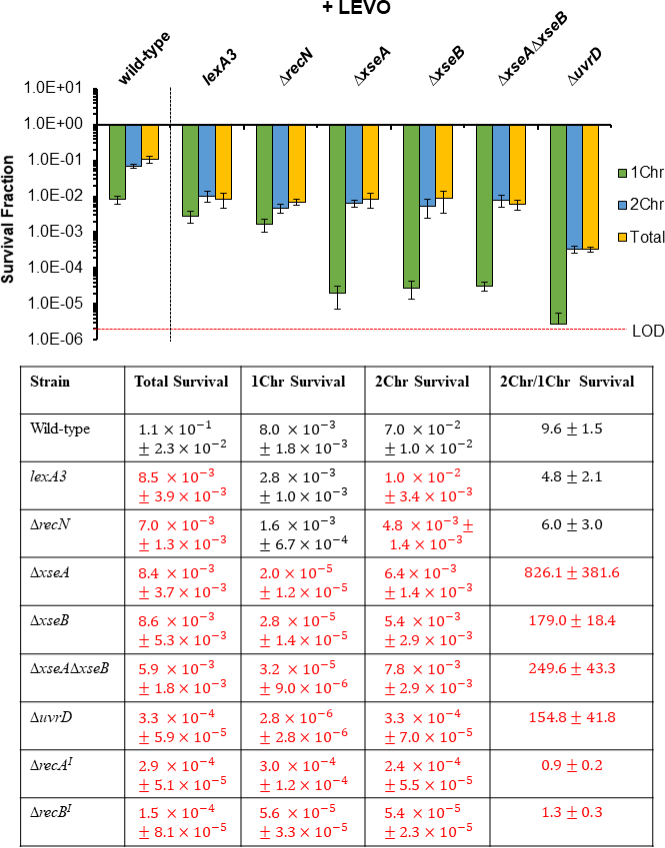
DNA repair mutants differentially impact 1Chr and 2Chr persister levels. Survival of sorted subpopulations in wild-type and DNA repair mutants after 5 h of treatment with 5 µg/mL LEVO. Data denote mean ± SEM for three or more biological replicates. Statistical analyses were conducted on the log-transformed survival after 5 h LEVO treatment on (i) 1Chr cells, (ii) 2Chr cells, (iii) total population, and (iv) fold-change in survival between 2Chr and 1Chr cells, respectively, using one-way analysis of variance followed by Tukey HSD *post hoc* tests [1Chr: *F*(6,22) = 50.7, *P* = 9.0e − 12; 2Chr: *F*(6,22) = 27.4, *P* = 3.8e − 9; total: *F*(6,22) = 25.7, *P* = 7.0e − 9; 2Chr/1Chr: *F*(6,22) = 18.9, *P* = 1.3e − 7]. Values in red denote statistical significance (adjusted *P* < 0.05) between indicated mutant strain and wild type using the same measurement (column name). *lexA3* and ∆*recN* showed significant declines in 2Chr survival when compared with wild-type 2Chr cells, whereas ∆*xseA*, ∆*xseB*, ∆*xseA*∆*xseB*, and ∆*uvrD* showed reductions in survival for both 1Chr and 2Chr cells, with a greater impact on 1Chr cells. ^I^Data from reference (41) based on sorting protocol with slight modifications from present study. Statistical analyses for ∆*recA* and ∆*recB* were performed following the same procedure as other mutant strains except that we compared them with the wild-type data obtained using the same sorting protocol [1Chr: *F*(2,10) = 26.0, *P* = 0.0001; 2Chr: *F*(2,10) = 65.9, *P* = 1.7e − 6; total: *F*(2,10) = 56.9, *P* = 3.5e − 6; 2Chr/1Chr: *F*(2,10) = 27.0, *P* = 9.2e − 5].

**Fig 5:**
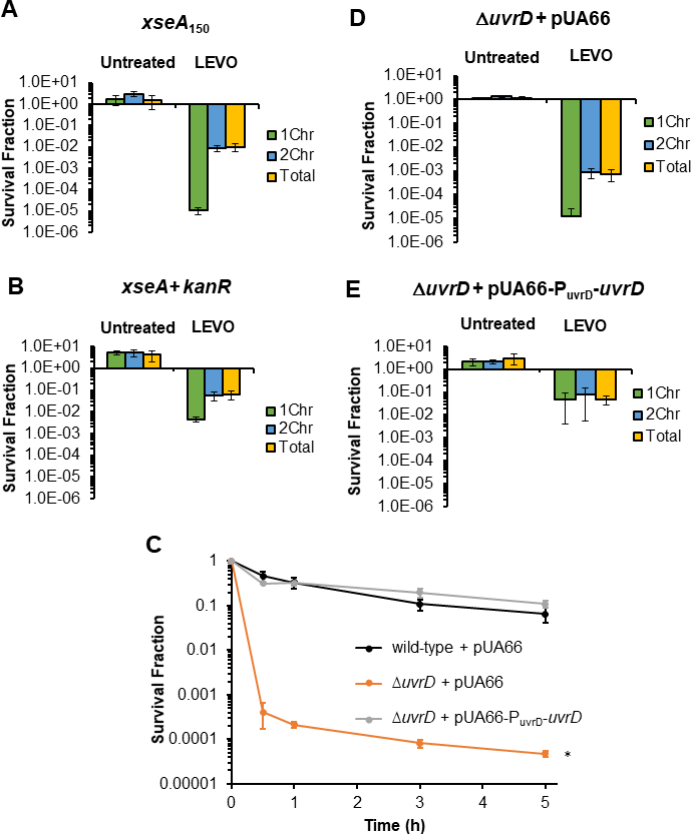
Complementation and genomic replacement restore FQ persistence to XseA and UvrD mutants. Survival of sorted subpopulations of (**A**) *xseA_150_*, (**B**) *xseA+ kanR*, (**D**) ∆*uvrD* + pUA66 (empty vector), and (**E**) *uvrD*-complemented strain following LEVO persister assay. Untreated controls denote samples treated with sterile water. (**C**) Survival of wild-type + pUA66, ∆*uvrD* + pUA66, and *uvrD*-complemented strain following the standard persistence assay protocol. Data denote mean ± SEM of two or more biological replicates. Statistical analyses were performed using one-way analysis of variance at 5 h FQ treatment time point followed by Tukey HSD post hoc test for multiple comparisons [*F*(2, 7) =146.5, *P* = 1.94e − 6]. Asterisk (⁠∗) denotes statistical significance (adjusted *P* < 0.05) in log-transformed survival between indicated mutant with plasmid and wild type with plasmid.

With XseA and XseB confirmed to be DNA repair proteins involved in LEVO and CIP persistence, we sought to assess whether their importance to persister survival, along with that of RecN, LexA, and UvrD, exhibited bias with respect to #Chr. To accomplish that, we assayed ∆*recN*, *lexA3*, ∆*uvrD*, ∆*xseA*, ∆*xseB*, and ∆*xseA*∆*xseB* for 1Chr and 2Chr persistence to LEVO in stationary-phase *E. coli* cultures (Fig. 4). Specifically, we stained stationary-phase cultures with Hoechst 33342, which is a nucleic acid stain that we confirmed previously to indicate #Chr in stationary-phase *E. coli* populations (41, 72), performed fluorescence-activated cell sorting (FACS) based on Hoechst 33342 staining, and then conducted FQ persistence assays on the sorted populations (Fig. S3). From these experiments, wild type exhibited an average of ~10-fold higher persister level for 2Chr compared to 1Chr cells (Fig. 4), which agrees well with previous observations (41). Consistent with normal persistence assays (not stained, not sorted), all mutants displayed lower persister levels than wild type in sorted populations (19, 20, 25, 32, 41–44). *lexA3* and ∆*recN* mutants showed significant declines in 2Chr persister levels, ~7- and ~15-fold compared to wild type, respectively, whereas 1Chr persister levels were similar to those of wild type (Fig. 4). These data suggested that SOS induction and RecN were important for 2Chr LEVO persistence and dispensable for 1Chr LEVO persistence. Interestingly, ∆*xseA*, ∆*xseB*, ∆*xseA*∆*xseB*, and ∆*uvrD* all showed significant declines in survival for both 1Chr and 2Chr persisters, but with much greater quantitative impacts on 1Chr LEVO persistence (Fig. 4). Specifically, ~400-, ~300-, ~250-, and ~3,000-fold reductions in persistence of 1Chr cells were observed for ∆*xseA*, ∆*xseB*, ∆*xseA*∆*xseB*, and ∆*uvrD,* respectively, which is in comparison to ~10-, ~10-, ~9-, and ~200-fold declines for 2Chr persisters when compared to the corresponding wild-type subpopulations. In other words, 1Chr cells were ~800-, 180-, 250-, and 150-fold less likely to be LEVO persisters compared to 2Chr cells, which far exceeded the relative persister level for wild type (~10-fold difference). Untreated samples indicated that declines in survival were due to LEVO treatments (Fig. S4). Pre-sort and post-sort controls (Materials and Methods) demonstrated that the sorting procedure did not impact persistence to LEVO appreciably (Fig. S5). Replacement of *xseA* via the genomic integration of *xseA* flanked by *kanR* into a nonfunctional mutant *xseA_150_* restored LEVO persister levels of 1Chr cells, 2Chr cells, and the total population to those observed for comparable subpopulations of wild type, whereas *xseA*_150_ demonstrated LEVO persister levels for those subpopulations that were comparable to ∆*xseA* (Fig. 5A and B; Fig. S6A and B). Complementation of ∆*uvrD* with *uvrD* expressed from its native promoter on a low copy plasmid restored LEVO persistence (Fig. 5C) (>400× MIC for ∆*uvrD*, Fig. S1). Sorting analyses further showed that 1Chr, 2Chr, and the total population persister levels of the *uvrD* complemented strain were comparable to the corresponding subpopulations of wild type, whereas an empty vector control exhibited LEVO persister levels that were comparable to ∆*uvrD* for those subpopulations (Fig. 5D and E; Fig. S6C and D). Together, these data revealed that ExoVII and UvrD are critical repair systems whose loss more significantly depressed persister levels of 1Chr cells following LEVO treatment, which is in comparison to RecA and RecB reported previously whose loss more significantly depressed the persistence of 2Chr cells (Fig. 4).

### UvrD and ExoVII are critical in the survival of 1Chr cells following CIP treatment

**Fig 6:**
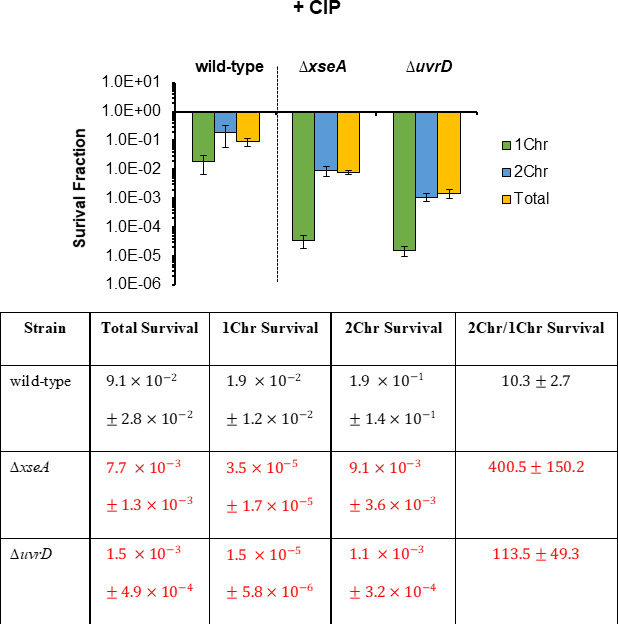
UvrD and ExoVII impact 1Chr survival more than that of 2Chr cells after CIP treatment. Survival of wild-type, ∆*xseA*, and ∆*uvrD* in 1Chr-sorted, 2Chr-sorted, and total-sorted subpopulations after 5 h of treatment with 1 µg/mL CIP. Data denote mean ± SEM of three or more biological replicates. Statistical analyses were conducted on log-transformed survival after 5 h CIP treatment of (i) 1Chr cells, (ii) 2Chr cells, (iii) total population, and (iv) fold-change in survival between 2Chr and 1Chr cells, respectively, using one-way analysis of variance followed by Tukey HSD *post hoc* tests. [1Chr: *F*(2,8) =48.1, *P* = 3.5e − 5; 2Chr: *F*(2,8) = 26.1, *P* = 0.0003; total: *F*(2,8) = 43.5, *P* = 5.0e − 5; 2Chr/1Chr: *F*(2,8) = 12.3, *P* = 0.003]. Values in red denote statistical significance (adjusted *P* < 0.05) between indicated mutant strain and wild type of the same #Chr. Both ∆*xseA* and ∆*uvrD* showed a greater impact on 1Chr survival than 2Chr survival.

To assess whether the importance of ExoVII and UvrD to 1Chr persistence is shared among the FQ antibiotic class, we used CIP as another clinically relevant FQ (27). In agreement with results for LEVO, persistence to CIP was far lower in both 1Chr and 2Chr subpopulations of Δ*xseA* and Δ*uvrD* when compared with the analogous wild-type subpopulations, and both quantitatively impacted 1Chr cells more than 2Chr cells (Fig. 6). Specifically, we observed ~500- and ~1,000-fold reductions in CIP persistence for 1Chr cells of Δ*xseA* and Δ*uvrD* with respect to analogous wild-type subpopulations, which is in comparison to ~20- and ~200-fold declines for 2Chr cells (Fig. 6). Pre-sort and post-sort controls again showed that neither Hoechst 33342 staining nor the time required for sorting impacted CIP persistence levels (Fig. S7).

### FQ persisters need ExoVII only when UvrD is present

**Fig 7:**
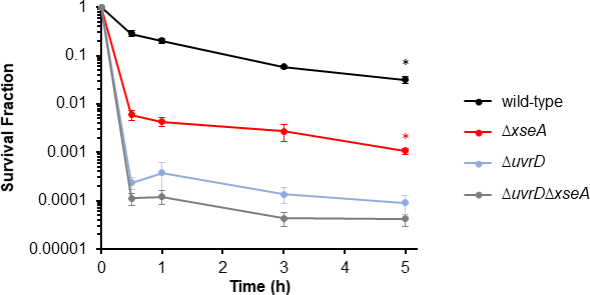
ExoVII is necessary for FQ persisters only in the presence of UvrD. Wild type, ∆*xseA*, ∆*uvrD*, or ∆*uvrD*∆*xseA* were grown to stationary phase as described in Materials and Methods and treated with 5 μg/mL LEVO. Data denote means ± SEMs of three or more biological replicates. Statistical analyses were performed using one-way analysis of variance assessing the effects of gene deletion(s) to log-transformed survival fraction after 5 h FQ treatment followed by Tukey HSD *post hoc* tests for multiple comparisons [*F*(3,18) = 39.5, *P* = 4.0e − 8]. Asterisk (⁠∗) denotes statistical significance (adjusted *P* < 0.05) between indicated strain and double-mutant ∆*uvrD*∆*xseA*.

To elucidate whether ExoVII and UvrD contribute to FQ persistence through the same pathway, we performed persistence assays on ∆*uvrD*, ∆*xseA*, and ∆*uvrD*∆*xseA* (Fig. 7). Data showed that ∆*uvrD*∆*xseA* exhibited comparable survival as ∆*uvrD* after 5-h LEVO treatment (<2-fold survival difference); whereas, ∆*uvrD*∆*xseA* exhibited a significant ~25-fold decline in survival compared to ∆*xseA* (Fig. 7). These data showed that with or without *xseA*, an impact of Δ*uvrD* was observed, whereas without *uvrD*, the impact of Δ*xseA* was absent. Since the impact of Δ*xseA* required *uvrD*, but the impacts of Δ*uvrD* did not require *xseA,* we postulate that XseA is involved in pathway(s) that use(s) UvrD, but UvrD is more broadly involved in survival than the pathway(s) that use XseA. Due to the multitude of functions UvrD is involved in, further work will be needed to dissect the different mechanisms of how it fully impacts FQ persistence.

## DISCUSSION

The original model of persistence attributed survival to dormancy and lack of corruption of antibiotic targets (34, 73, 74). However, a number of groups have demonstrated that persistence is not a one-size-fits-all phenomenon, and that it often depends on the antibiotic and environment (19, 22, 33, 43, 75–81). FQ persisters are a prime example of such heterogeneity because they do not survive due to lack of antibiotic-induced damage, but survive by repairing it (33, 43). In *E. coli*, FQs kill mainly by disturbing the function of DNA gyrase, leading to DSBs at saturating concentrations (41, 62). Based on the mechanism of action of FQs, we reasoned that the characteristics of FQ-induced DNA damage and the ensuing DNA repair activities are the two key processes that influence persistence to this drug class. Using genome-wide mapping of FQ-stabilized gyrase cleavage sites (GCSs) in growth-inhibited *E. coli* populations, we showed that the extent of DNA damage was an extremely strong predictor of FQ persistence (24). On the DNA repair side, our work and that of others have demonstrated SOS induction in FQ persisters, which helps explain why numerous DNA repair mutants have impaired FQ persistence levels (19, 20, 25, 33, 41, 43, 44). Recently, our work has expanded that knowledge base by identifying at least two distinct FQ persister subpopulations in isogenic cultures: persisters arising from cells with 2Chr that are HR-proficient; and persisters arising from cells with 1Chr that are HR-impaired (41). In that study, the initial size, rate of volume expansion, and time of division all significantly differed between those FQ persister subtypes (41). Furthermore, it was shown that ∆*recA* and ∆*recB* reduced 2Chr persistence by ~100-fold, in comparison to ~10-fold declines in 1Chr persistence, which demonstrated that the importance of DNA repair systems to FQ persisters will depend on the single-cell #Chr (41).

To better understand how the usage of DNA repair machinery varies between 1Chr and 2Chr FQ persisters, we performed an investigation of DNA repair mutants that we and others have shown to influence FQ persistence in stationary-phase cultures (19, 20, 25, 32, 41–44). In addition, we examined the role of ExoVII (XseA and XseB) in LEVO and CIP persistence due to recent evidence suggesting that it can process DNA breaks left from FQ treatment (59), and a recent study that observed that Δ*xseA* and Δ*xseB* decreased tosufloxacin persistence (45). When investigating the impacts of repair mutants as a function of #Chr, we observed that SOS induction and RecN were important to 2Chr persistence (Fig. 4). In consideration that RecN is a cohesion factor that holds together sister chromosomes for HR, its significant impact on 2Chr persisters and lack of impact on 1Chr persisters agree well with its documented function (51). LexA on the other hand controls DNA repair genes that are involved in HR as well as other DNA repair pathways that do not require a secondary template chromosome, such as NER (82). The lack of significant impacts of *lexA3* on 1Chr FQ persistence suggests that induction of SOS genes does not foster survival of 1Chr persisters. Alternatively, our data suggested that while both subunits of ExoVII (XseA and XseB) and UvrD decreased persistence at the total population level (Fig. 1 and 7), their impacts on 1Chr persistence were far greater than their impacts on 2Chr persistence (Fig. 4). Specifically, ~800-, ~200-, ~200-, and ~200-fold differences were observed between 1Chr and 2Chr persister levels for ∆*xseA*, ∆*xseB*, ∆*xseA*∆*xseB*, and ∆*uvrD*, respectively, when compared to ~10-fold difference for wild type when LEVO was used for treatment. Furthermore, we showed that these findings were generalizable to CIP using Δ*xseA* and Δ*uvrD* (Fig. 6), even though CIP and LEVO have been shown to exhibit both shared and unique GCSs with varying cleavage strengths (24).

ExoVII has been implicated in both repair and recombination pathways, albeit usually in a redundant fashion with other exonucleases (68, 71). For example, exponentially growing ∆*xseA* mutants were found to be modestly sensitive to UV irradiation as shown by a decline in colony-forming units (CFU) after exposure (83, 84), and incubation of Δ*xseA* on plates with nalidixic acid showed decreased survival (84). However, a ∆*xseA*∆*recJ* double mutant, of which both XseA and RecJ have the ability to degrade ssDNA in the 5´- 3´ direction, was extremely sensitive to UV (85). Indeed, ssExos, including ExoVII, function redundantly to repair UV damage, with mutants deficient in three or four ssExos most susceptible to UV light (70, 86). Similarly, ExoI, RecJ, ExoVII, and ExoX act redundantly during MMR to degrade ssDNA liberated by UvrD following incision by MutH (68, 85, 87, 88). Here, we showed that ExoVII has a distinctive role in processing damage induced by FQs in persisters, which cannot be compensated for by other exonucleases because individual deletion of *xseA* or *xseB* leads to pronounced declines in FQ persistence (Fig. 1). We postulate that ExoVII contributes non-redundantly to FQ persistence through its ability to process DNA ends with tyrosyl adducts on 5´ phosphate overhangs (59). However, the question remains as to why ExoVII is more important to 1Chr persisters than 2Chr persisters considering that 5´ tyrosyl residues would need to be removed for both 1Chr and 2Chr cells. Though it has yet to be explored what machinery HR-proficient persisters use to address such DNA adducts in the absence of ExoVII, the extremely low and indistinguishable survival between ∆*xseA*∆*uvrD* and ∆*uvrD* following FQ treatment suggested that ExoVII is only important when UvrD is present.

We demonstrated that UvrD is important to both 1Chr and 2Chr persisters, with a far greater impact on 1Chr compared to 2Chr cells. Our data showed a >200-fold drop in 2Chr persistence and >2,800-fold drop in 1Chr persistence compared to wild type. At such levels, ∆*uvrD* 1Chr persisters were near the limit of detection for our persistence assays, and we postulate that ∆*uvrD* 1Chr cells may not be able to survive FQ-induced damage. Interestingly, our data suggested that the survival of the 2Chr subpopulation in ∆*uvrD* was significantly lower than all the other DNA repair mutants tested here, including ExoVII mutants, and slightly higher to what was observed with ∆*recA* and ∆*recB* (41), which would argue that 2Chr persisters use UvrD, RecA, and RecB to survive. UvrD, together with the UvrABC nuclease complex, constitute key mediators of NER (89), and UvrD also plays roles in MMR, recombination, replication restart, and resolution of Holliday junctions (54, 57). Interestingly, UvrD has been shown to disrupt RecA-ssDNA filaments (56, 90), and loss of *uvrD* has been observed to modestly increase (~5-fold) basal expression of the SOS response (91). Furthermore, UvrD plays a role in antirecombination where it is directed by MutS and MutL to mismatches during RecA-mediated strand exchange where it then unwinds those intermediates with its helicase activity (58). Recent work using time-lapse microscopy showed that persisters of Δ*uvrD* took longer to resume growth compared to wild type following FQ treatment, and that the double-deletion strain Δ*uvrD*Δ*mfd*, which is deficient in transcription-coupled repair, exhibited higher persister levels than Δ*uvrD* alone (20). In a different study, inhibition of transcription or translation during the post-FQ recovery period increased the abundance of persisters, and the phenomenon was shown to exhibit epistasis between *recA* and *uvrD*, where it was observed in both single-deletion strains, but not the double-deletion mutant (92). In consideration of the multitude of functions performed by UvrD and its interactions with RecA, further work to elucidate the mechanistic details of how UvrD exerts its impact on FQ persistence will be needed.

In conclusion, we have identified DNA repair proteins that impact FQ persister levels differently based on the number of chromosomes contained in individual bacteria. Data reported here suggest that XseA, XseB, and UvrD are critically important DNA repair proteins for HR-impaired 1Chr persisters, whereas SOS induction and RecN are more important for HR-proficient 2Chr persisters. This adds to knowledge from a previous study where RecA and RecB were important to both 1Chr and 2Chr persisters, but their quantitative impacts on 2Chr persistence were far greater (41). Analogously, the ability of mutants deficient in UvrD or XseAB to decrease FQ persister levels in both 1Chr and 2Chr subpopulations make them intriguing targets for future drug development, of which XseAB inhibitors have already begun to be identified (59).

## MATERIALS AND METHODS

### Bacterial strains and plasmids

All strains and plasmids used in this study are listed in Table S1 (32,93,94,95). *E. coli* strain MG1655 was used as wild type. All other strains used in the experiments, unless indicated, were derived from MG1655. DNA oligonucleotides used for cloning and sequence verification are listed in Table S1. DNA repair mutants used in this work were generated by P1 transduction (96) from the corresponding mutant in the Keio collection (97). Where indicated, *kanR* was removed using pCP20 following steps as previously described (98, 99). All sequences were confirmed by PCR and sequencing (Genewiz, South Plainfield, NJ).

All plasmids were constructed by Gibson Assembly (NEB Gibson Assembly Cloning Kit) using the primers listed in Table S1, and verified by sequencing (Genewiz, South Plainfield, NJ). For Δ*xseA*, we initially tried to complement *xseA* via plasmid-borne expression under both its native promoter and an inducible (P*_T5_*) promoter. Both attempts yielded partial complementation of survival when using LEVO. Previous studies suggested that *xseA* overexpression could impair ExoVII activity (66) or be toxic to cells (67), which could explain the partial complementation we observed here with plasmid-based techniques. Due to this technical challenge, we opted to genomically replace *xseA*. We first integrated a *kanR* cassette flanked by flippase recognition target (FRT) sites immediately downstream of the 150th amino acid of XseA in place of amino acid 151 to 456 (right before the stop codon). We named this strain MG1655 *xseA*_150_ FRT-*kanR*-FRT. We then cured that strain to create MG1655 *xseA*_150_, which was used as a truncated nonfunctional *xseA* throughout the study. We subsequently integrated the remainder of the *xseA* sequence flanked by a *kanR* cassette (to form MG1655 *xseA+ kanR*), or that sequence containing a D155A mutation, into *xseA*_150_ strain using the method of Datsenko and Wanner (98). MG1555 *xseA*(F63A) *kanR* was generated by integrating a PCR product with the *xseA* sequence carrying a F63A mutation flanked by *kanR* into MG1655 ∆*xseA* using the method of Datsenko and Wanner (98). All constructs were confirmed by sequencing. We note that the only difference between MG1655 *xseA+ kanR*, MG1555 *xseA*(F63A) *kanR*, and MG1555 *xseA*(D155A) *kanR* are the intended mutations. Colonies were selected on 25 µg/mL kanamycin (KAN) and verified via sequencing of PCR products and mass spectrometry (see below).

### Reagents

All components, chemicals, and antibiotics used in this study were purchased from Sigma Aldrich (St. Louis, MO) or Fisher Scientific (Waltham, MA), unless otherwise noted. All enzymes were purchased from New England Biolabs. In all experiments, autoclaved MilliQ water (18.2 MΩ·cm at 25°C, Merck Millipore Ltd, Burlington, MA) was used as solvent unless otherwise indicated. Luria-Bertani (LB) medium (Fisher Scientific) was prepared from individual components using 10 g/L tryptone, 5 g/L yeast extract, and 10 g/L NaCl, and LB agar was prepared with 25 g/L BD Difco pre-mixed LB Miller broth and 15 g/L agar. The media and agar were then autoclaved at 121°C for 30 min to achieve sterilization. M9 glucose media was prepared using autoclaved 5× M9 minimal salts (33.9 g/L Na_2_HPO_4_, 15 g/L KH_2_PO_4_, 5 g/L NH_4_Cl, 2.5 g/L NaCl), 0.1 mM CaCl_2_, 2 mM MgSO_4_, and 10 mM glucose as the sole carbon source. M9 glucose media was filter-sterilized using a 0.22 µm filter (Merck Millipore Ltd, Burlington, MA) upon preparation.

For plasmid maintenance, AMP and KAN were used at working concentrations of 100 µg/mL and 50 µg/mL, respectively, and sterile filtered using 0.22 µm filter (Merck Millipore Ltd, Burlington, MA). For mutant selection on plates, the appropriate antibiotic(s) were added into LB agar after it cooled down to about 50°C. For FQ persistence assays and sorting, stock solutions of 5 mg/mL LEVO in 20 mM NaOH and 1 mg/mL CIP in 0.2 M HCl were prepared and filter-sterilized using 0.22 µm filters (Merck Millipore Ltd, Burlington, MA). All stock solutions were prepared fresh for each experiment and sterile filtered through a 0.22 µm filter (Merck Millipore Ltd, Burlington, MA). A working concentration of 5 µg/mL LEVO and 1 µg/mL CIP were used in persistence assays and sorting experiments. The working concentrations were selected to be in the regime of survival versus antibiotic concentration plots where further increases in antibiotic concentration fail to increase killing appreciably (24). Hoechst 33342 (ThermoFisher) stock was diluted 1:10 and cells were stained at a final concentration of 5 µg/mL.

### MIC testing

Test tubes filled with 2 mL LB were inoculated with bacterial stocks stored in 25% glycerol at −80°C and incubated for ~20 h overnight at 37°C with shaking. After overnight incubation, 1 mL aliquots were centrifuged in microcentrifuge tubes for 3 min at 15,000 rpm, and cell pellets were resuspended in 1 mL of sterile 0.85% NaCl solution. The cells were then diluted to an optical density (OD_600_) ~0.2 in 1 mL of sterile 0.85% NaCl. A sterile cotton swab was dipped into the cell solution, and cells were streaked onto Mueller Hinton agar plates by rapidly swiping back and forth to cover the entire surface of the plate with bacteria. The plate was rotated 60°, the cotton swab was dipped into the cell solution again, and cells were swiped onto the surface of the agar again as described. This was completed one more time after rotating the plate another 60°. The plates were allowed to dry at room temperature before E-test strips (bioMérieux, Marcy-l’Étoile, France; Liofilchem Inc, Roseto degli Abruzzi, Italy) were aseptically placed into the center of the agar using ethanol- and flame-sterilized tweezers. Plates were incubated at 37°C for 16 h, and the MIC was determined to be the point at which the ellipse clearing intersected with the E-test strip. Averages represent three biological replicates.

### SDS-PAGE and mass spectrometry analysis

To validate the identity of complemented and catalytic mutant *xseA* strains, cells were inoculated from glycerol stocks to 2 mL LB media and incubated overnight at 37°C with shaking at 250 rpm. One-milliliter aliquots were removed from each sample and centrifuged in microcentrifuge tubes for 3 min at 15,000 rpm. Supernatant were removed, cell pellets were resuspended 1 mL MilliQ water, and cells were centrifuged again. Supernatant were removed, and cell pellets were resuspended in 50 µL H_2_O and transferred into PCR tubes. Samples were then mixed with equal volumes of 2× Laemmli sample buffer (Bio-Rad, Philadelphia, PA), loaded onto SDS-PAGE gels (Mini-PROTEAN Precast Protein Gels, Bio-Rad, Philadelphia, PA), and run at 150V for 60 min following manufacturers’ instructions. Gels were carefully removed from the apparatus, gently shaken in MilliQ water for 15 min, and stained overnight in Coomassie blue stain [50% (vol/vol) methanol, 0.05% (wt/vol) Coomassie brilliant blue R-250 (BioRad), 10% (vol/vol) acetic acid, and 40% H_2_O] with gentle shaking. The following morning, Coomassie blue stain was removed, and gels were boiled twice in ~500 mL MilliQ water in the microwave for 8 min, changing the water after each boil, in order to remove excess stain. Gels were then further destained by an overnight gentle shaking in destaining buffer [40% (vol/vol) methanol, 10% (vol/vol) acetic acid, and 50% H_2_O]. Destaining buffer was removed, and gels were rinsed three times in MilliQ water before imaging.

Gel bands were excised surrounding the expected size of XseA and its catalytic and DNA binding mutants (~52 kDa) or XseA_150_ (~37 kDa) (65) for mass spectrometry analyses (Proteomics & Mass Spectrometry Facility, Princeton, NJ) following the protocol described previously in reference (24) with slight modifications. Briefly, an easy-nLC 1200 UPLC system coupled with an Orbitrap Fusion Lumos (Thermo Scientific, USA) was used for analysis. Two microliters of samples (~360 ng) was injected per run and loaded directly onto a 45 cm-long, 75 µm inner diameter nanocapillary column packed with 1.9 µm C18-AQ (Dr. Maisch, Germany). Column temperature was set at 45°C and 2-h gradient method with a flow rate of 300 nL/min (mobile phase compositions: 0.1% formic acid in water and 0.1% formic acid in 80% acetonitrile/water). The mass spectra were collected in data-dependent mode. Mass spectrometry (MS) scan (positive mode, profile data type, automatic gain control (AGC) 4e5, maximum injection time (IT) of 54 ms, 375–1,500 *m*/*z*, 120,000 resolution) in the Orbitrap was followed up by high energy collisional dissociation (HCD) fragmentation in the ion trap with 35% collision energy (AGC 1e4, maximum IT of 54 ms, minimum of 5,000 ions). Previously fragmented peptides were prevented from repeated fragmentation for 60 s.

Carbamidomethylation of cysteine was used as a fixed modification, oxidation of methionine, deamidation of asparagine, and glutamine were specified as dynamic modifications. Pyroglutamate conversion from glutamic acid and glutamine were set as dynamic modifications at peptide N-terminus. Acetylation was specified as dynamic modification at protein N-terminus. A maximum of two missed trypsin cleavages were allowed. Files were searched against UP000000558 *Escherichia coli* database downloaded from Uniprot.org. Relevant point mutations of ExoVII were added to database.

Scaffold (version Scaffold_4.11.1, Proteome Software Inc., Portland, OR) was used for protein identifications. Peptide identifications were accepted if they could be established at greater than 95.0% probability by the Scaffold Local False Discovery Rate (FDR) algorithm. Protein identifications were accepted if they could be established at greater than 99.9% probability and contained at least two identified peptides. Protein probabilities were assigned by the Protein Prophet algorithm (100).

The mass spectrometry analysis yielded peptide sequences that verified the mutations of interest in peptides covering 17% of XseA D155A (Fig. S8). Peptide fragments were also obtained for XseA F63A, suggesting that the mutant protein was produced by the cells, yet the peptide fragments that would have contained the amino acid substitution of interest were not observed (Fig. S8). MG1655 ∆*xseA* and MG1655 *xseA*_150_ did not produce any peptides mapping to XseA. We note that all mutations were verified by sequencing.

### FQ persistence assays

Cells were inoculated from frozen 25% glycerol stocks stored at −80°C into test tubes filled with 2 mL LB supplemented with antibiotics as needed for selection and cultured for 4–5 h at 37°C with shaking at 250 rpm. The OD_600_ of the strains were then measured using a spectrophotometer, and aliquots of cells were pipetted into microcentrifuge tubes and centrifuged for 3 min at 15,000 rpm. Supernatants were removed, and cells were resuspended in 300 µL of M9 glucose media, followed by inoculation to an OD_600_ ~0.01 into 25 mL of M9 minimal media (with antibiotic for plasmid retention as needed) in 250 mL baffled flasks. The cultures were grown overnight for 20 h at 37°C with shaking at 250 rpm. After 20 h, cultures were treated with 5 µg/mL LEVO, 1 µg/mL CIP, or an equal volume of solvent for untreated cultures, and incubated at 37°C for 5 h with shaking. For the assays with LEVO that were performed over a range of concentrations in Fig. S1, 50 μL of solvent or 500× LEVO stocks were used to achieve final concentrations of 0, 0.1, 0.5, 1, 5, and 10 µg/mL LEVO. Right before the addition of FQ and at 0.5, 1, 3, and 5 h following treatments, 500 µL samples were removed and washed three times. For each wash, the cells were centrifuged for 3 min at 15,000 rpm followed by the removal of supernatant and resuspension into 500 µL phosphate-buffered saline (PBS). After the last wash, serial fivefold dilutions were performed, and 10 µL aliquots of each dilution were plated on LB agar. Plates were incubated at 37°C for 20–24 h for CFU enumeration.

### FACS

#### Sample preparation for #Chr sort

Figure S3 provides a simplified schematic of the FACS procedure performed to quantify FQ persistence with respect to #Chr. Cells were grown to stationary phase following the same protocol as the preceding section. Following 20 h incubation at 37°C with shaking in M9 glucose media and prior to any FQ treatment, two 500 µL samples were removed from each flask and centrifuged for 3 min at 15,000 rpm. Supernatants were removed followed by resuspension in 500 µL PBS (the live cell sample) or 500 µL 4% paraformaldehyde (PFA) for 15 min at room temperature (the fixed sample). Both samples were centrifuged for 3 min at 15,000 rpm and resuspended in 500 µL PBS. Samples were diluted to an OD_600_ ~0.025–0.03 in 5 mL PBS prefilled in a 15 mL conical tube and either stained with 5 µg/mL Hoechst 33342 at 37°C in the dark for ~30 min or remained unstained. After staining, cells were transferred to flow cytometry tubes for FACS analysis.

Single cells were sorted based on Hoechst 33342 fluorescence intensity as determined by FACSDiva version 8.0 software and using a BD Biosciences FACSAria Fusion Special Order Research Product (San Jose, CA). Gating on forward scatter (FSC) and side scatter (SSC) enabled the identification of cells as compared to blank control. Cells were gated based on SSC height and width (SSC-H and SSC-W), followed by gating with Hoechst33342-W and Hoechst33342-A, which enabled the exclusion of cell doublets. Single-cell Hoechst 33342 fluorescence values were acquired by exciting each cell with a 355 nm laser run at 60 mW power (Coherent, Santa Clara, CA) and followed by using a 410 nm long pass filter and a 450/50 nm bandpass filter. Samples were analyzed using FCS Express Software version 6 Research Use Only (*De Novo* Software, Glendale, CA). One million single cells were sorted using single-cell mode, a high purity sorting method that sacrifices yield and time to obtain higher purity. A previous study demonstrated nearly 99% purity using the single-cell sorting mode (41). Detailed methodologies to segregate bacterial populations based on #Chr were previously described (72).

#### Persistence assay following #Chr sort

One million single cells were sorted into 1 mL sterile-filtered spent media based on chromosome number (1Chr and 2Chr) at 37°C, bringing the total volume to 2 mL. Additionally, a total unsegregated population (Tot) was also collected. Each sorted sample was then equally divided into two 14 mL polypropylene test tubes (1 mL each), which corresponded to the drug-treated sample and an untreated control. Samples were treated with 5 µg/mL LEVO, 1 µg/mL CIP, or equal volume of solvent and incubated for 5 h at 37°C with shaking at 250 rpm. Right before treatment, 100 µL of cell suspensions were removed from each sample and added to microcentrifuge tubes prefilled with 900 μL sterile PBS (first wash). Those samples were then centrifuged for 3 min at 15,000 rpm, 900 µL of supernatants were removed, and 900 µL of fresh PBS were added (second wash). This process was repeated for a third wash. After the third wash in PBS, cells were centrifuged for 3 min at 15,000 rpm, and 900 µL of supernatant were removed. Cell pellets were resuspended in the remaining 100 µL of PBS, and 20 µL of cells were added to 80 µL of PBS in 96-well plates. Fivefold serial dilutions were performed and 10 µL of dilutions were plated onto LB agar plates for CFU enumeration.

After 5 h of treatment, 100 µL of cells were removed from each sample washed three times and serially diluted in 96-well plates as described above. For untreated cells, 10 µL of wells 1–6 were plated on LB agar and allowed to dry. For treated samples, 50 µL of the undiluted samples, as well as wells 1–3, were plated onto LB agar and allowed to dry. Additionally, for treated samples, the remaining ~800 µL of cells were added to an empty microcentrifuge tube, centrifuged for 3 min at 15,000 rpm, 700 µL of supernatant was removed, and cells were subsequently washed three times in 900 µL PBS as described. After the final wash, cells were centrifuged and all but 100 µL of supernatant were removed. The cells were resuspended, and the remaining 100 µL of cells were plated onto LB agar using sterile glass spreaders, which had been ethanol- and flame-sterilized beforehand. Plates were incubated for 20–24 h at 37°C for CFU enumeration.

#### Controls for #Chr sort

Pre-sort and post-sort controls were conducted as previously described (41) to ensure that neither the sorting process itself nor the time required to sort cells impacted persistence measurements. Briefly, for the pre-sort controls, stained and unstained cells that had been prepared for sorting in the previous section were further diluted to an OD_600_ <0.001 in 2 mL of 50% PBS and 50% sterile spent media. Each sample was then equally divided into two, by pipetting 1 mL into two 14 mL polypropylene tubes for treated and untreated conditions. One hundred microliters of cell suspensions were removed and added to microcentrifuge tubes prefilled with 900 µL PBS, washed, and plated as described above. Cultures were treated with 5 µg/mL LEVO, 1 µg/mL CIP, or equal volume solvent and incubated for 5 h at 37°C with shaking at 250 rpm. After 5 h of treatment, samples were processed identically to those described above. For the post-sort controls, the same procedure was followed, except that the cells were sampled from cell suspensions of stained and unstained cells that had not been sorted after the sorting procedure was done.

### Statistical analysis

At least three independent biological replicates were performed for each experiment unless indicated otherwise. Error bars represent SEM. Significance was assessed using one-way analysis of variance followed by Tukey’s *post hoc* tests on log-transformed survival and a *P*-value threshold of 0.05.

## SUPPLEMENTAL MATERIAL

Figure S1 mbio.00374-24-s0001.tif MICs of mutant strains and LEVO persistence assays performed at varying concentrations.

Figure S2 mbio.00374-24-s0002.tif Untreated controls for persistence assays with mutants.

Figure S3 mbio.00374-24-s0003.tif Schematic of experiments to measure persistence of subpopulations with different #Chr.

Figure S4 mbio.00374-24-s0004.tif Untreated controls of sorted 1Chr, 2Chr, and total populations.

Figure S5 mbio.00374-24-s0005.tif Unsorted pre-sort and post-sort persister assay controls for DNA repair mutant FACS experiments.

Figure S6 mbio.00374-24-s0006.tif Controls for complementation and genetic replacement FACS experiments.

Figure S7 mbio.00374-24-s0007.tif Unsorted pre-sort and post-sort persister assay controls for DNA repair mutant FACS experiments following CIP treatment.

Figure S8 mbio.00374-24-s0008.tif Confirmation of *xseA* mutants by mass spectrometry.

Table S1 mbio.00374-24-s0009.docx Strains, plasmids, and primers.

ASM does not own the copyrights to Supplemental Material that may be linked to, or accessed through, an article. The authors have granted ASM a non-exclusive, world-wide license to publish the Supplemental Material files. Please contact the corresponding author directly for reuse.
