# Supplementary figures and images for: Differential impacts of DNA repair machinery on fluoroquinolone persisters with different chromosome abundances
Source: mBio. 2024 Apr 2;15(5):e00374-24. doi: 10.1128/mbio.00374-24 (PMC11077951; doi:10.1128/mbio.00374-24)

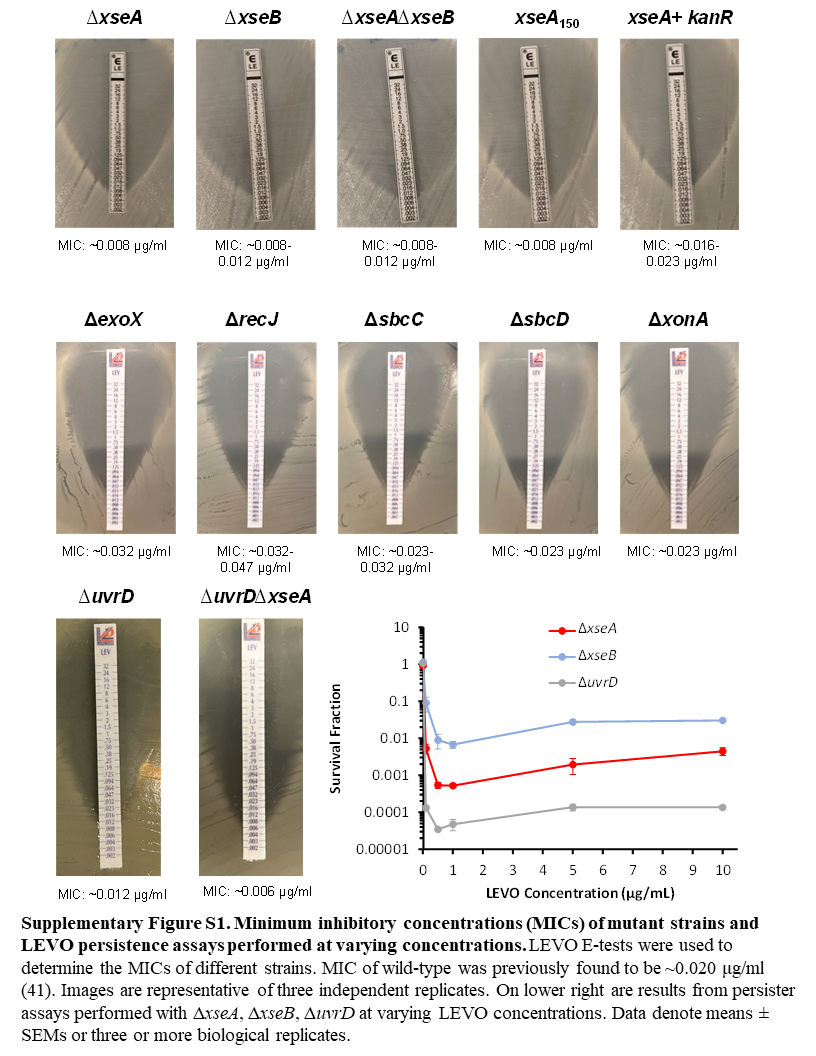

Supplement: Figure S1 — MICs of mutant strains and LEVO persistence assays performed at varying concentrations. [file mbio.00374-24-s0001.tif]

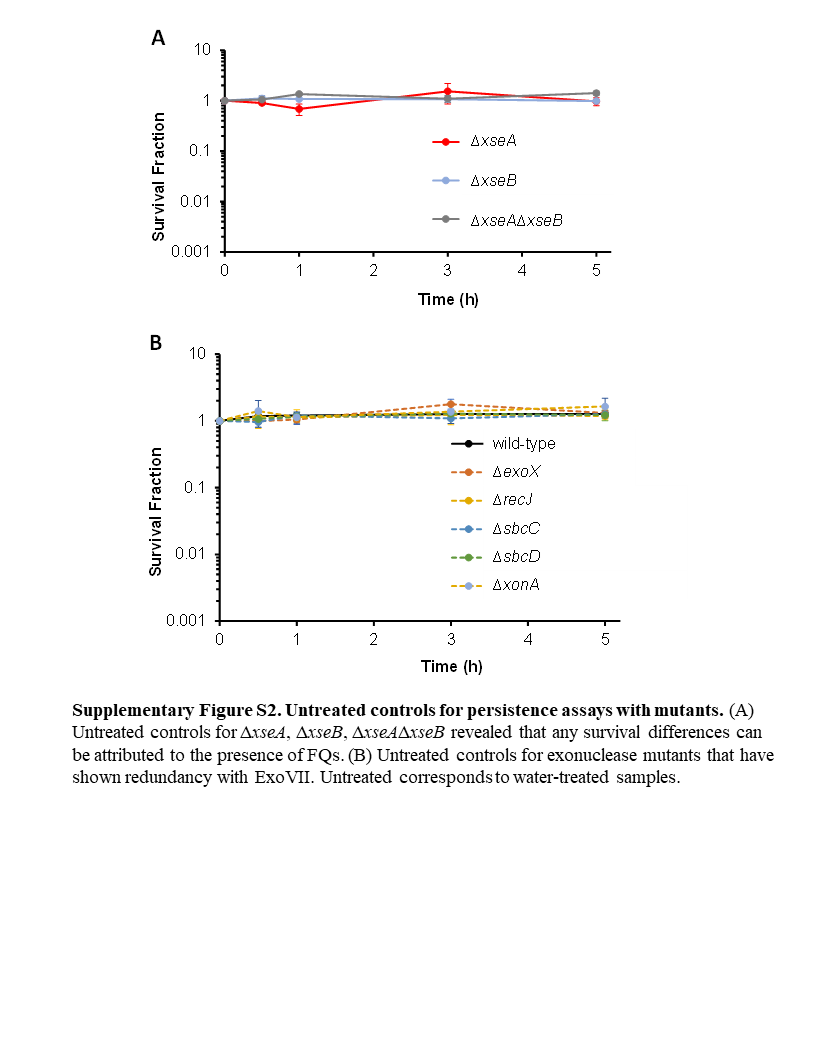

Supplement: Figure S2 — Untreated controls for persistence assays with mutants. [file mbio.00374-24-s0002.tif]

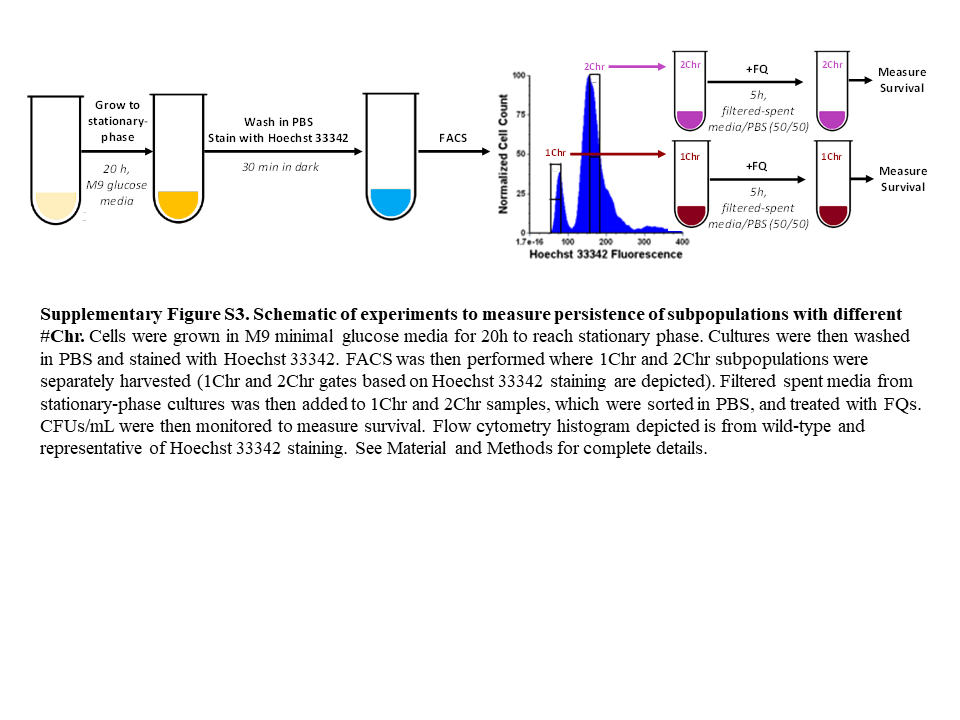

Supplement: Figure S3 — Schematic of experiments to measure persistence of subpopulations with different #Chr. [file mbio.00374-24-s0003.tif]

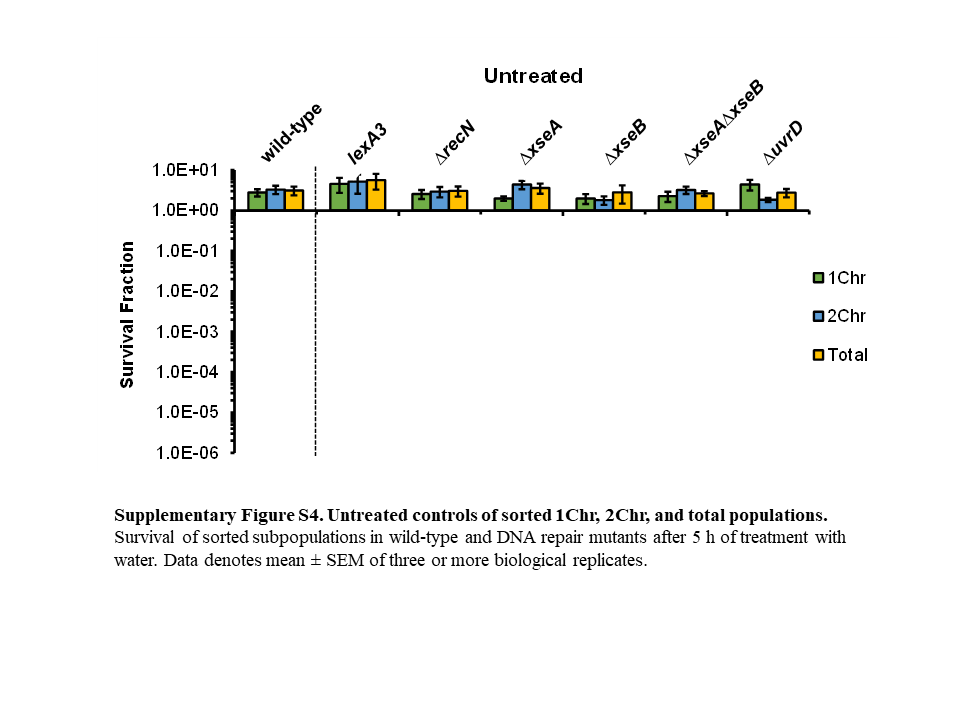

Supplement: Figure S4 — Untreated controls of sorted 1Chr, 2Chr, and total populations. [file mbio.00374-24-s0004.tif]

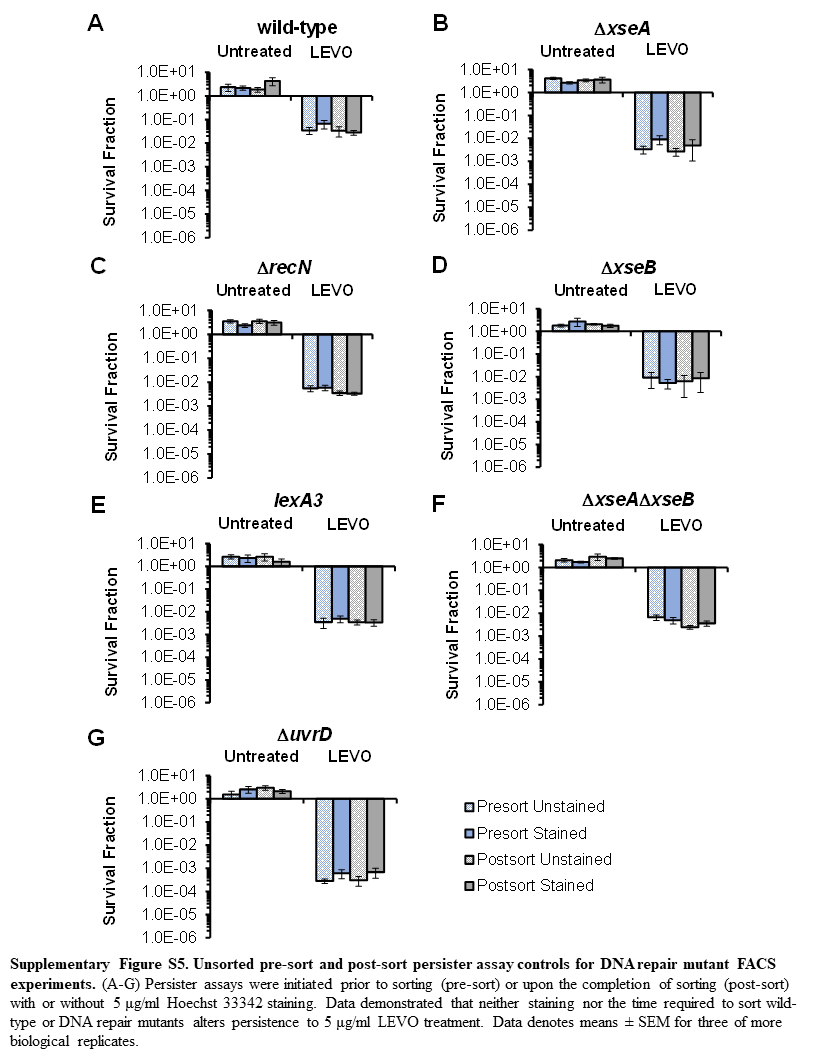

Supplement: Figure S5 — Unsorted pre-sort and post-sort persister assay controls for DNA repair mutant FACS experiments. [file mbio.00374-24-s0005.tif]

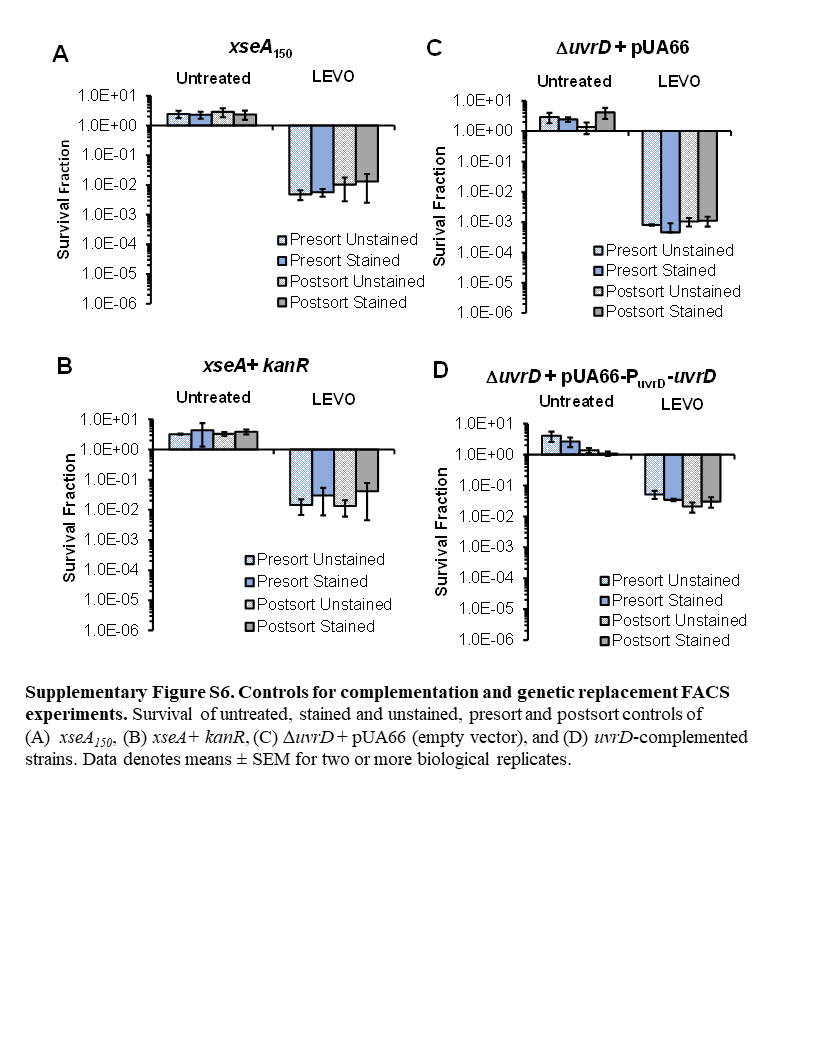

Supplement: Figure S6 — Controls for complementation and genetic replacement FACS experiments. [file mbio.00374-24-s0006.tif]

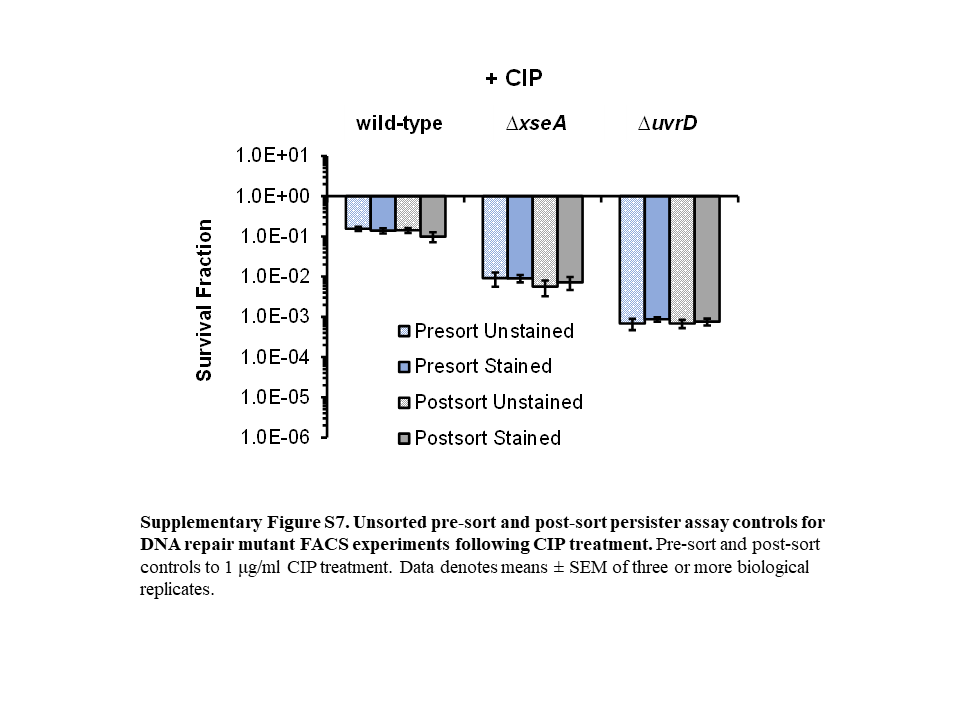

Supplement: Figure S7 — Unsorted pre-sort and post-sort persister assay controls for DNA repair mutant FACS experiments following CIP treatment. [file mbio.00374-24-s0007.tif]

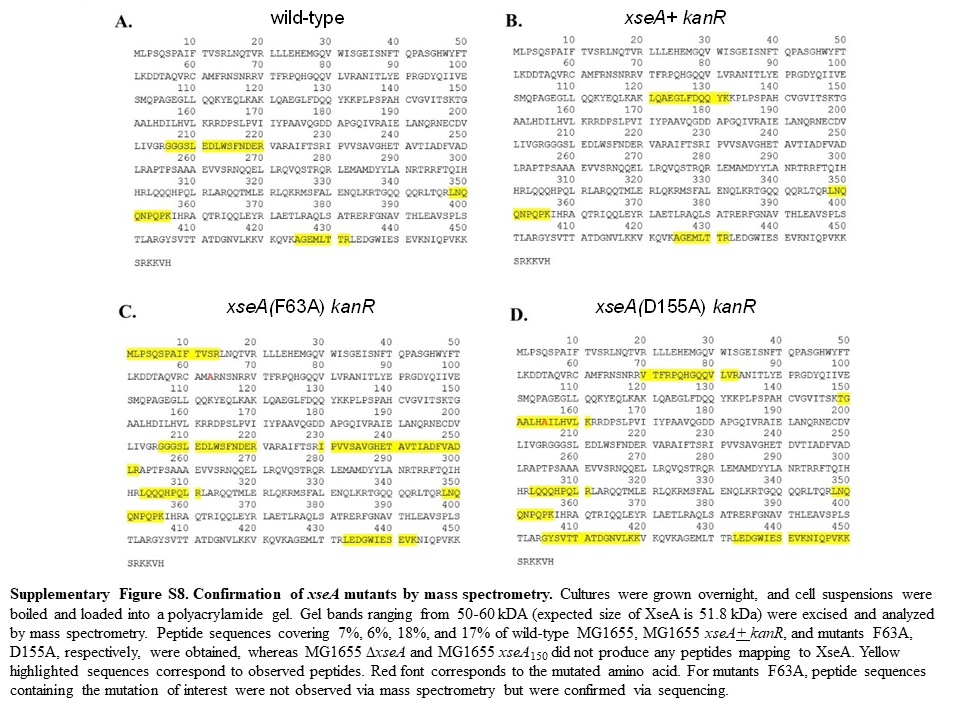

Supplement: Figure S8 — Confirmation of xseA mutants by mass spectrometry. [file mbio.00374-24-s0008.tif]
