# Supplementary material for: Differential impacts of DNA repair machinery on fluoroquinolone persisters with different chromosome abundances
Source: mBio. 2024 Apr 2;15(5):e00374-24. doi: 10.1128/mbio.00374-24 (PMC11077951; doi:10.1128/mbio.00374-24)
Supplement: Table S1 — Strains, plasmids, and primers. [file mbio.00374-24-s0009.docx]

Supplementary Table S1. Bacterial strains, plasmids, and primers

| **Strain** | **Genotype** | | | **Source or Reference** | | |
| --- | --- | --- | --- | --- | --- | --- |
| MG1655 | F^-^ λ^-^ *ilvG*^-^ *rfb*-50 *rph*-1 | | | ATCC 700926 (98) | | |
| AM02 | MG1655 Δ*xseA* | | | This work | | |
| AM03 | MG1655 Δ*xseB* | | | This work | | |
| AM04 | MG1655 Δ*xseA*Δ*xseB* | | | This work | | |
| AM05 | MG1655 *xseA*_150_ | | | This work | | |
| AM06 | MG1655 *xseA*+ *kanR* | | | This work | | |
| AM07 | MG1655 *xseA*(F63A) *kanR* | | | This work | | |
| AM08 | MG1655 *xseA*(D155A) *kanR* | | | This work | | |
| AM09 | MG1655 Δ*uvrD* | | | This work | | |
| AM10 | MG1655 Δ*exoX* | | | This work | | |
| AM11 | MG1655 Δ*xonA* | | | This work | | |
| AM12 | MG1655 Δ*sbcC* | | | This work | | |
| AM13 | MG1655 Δ*sbcD* | | | This work | | |
| AM14 | MG1655 Δ*recD* | | | This work | | |
| AM15 | MG1655 Δ*recJ* | | | This work | | |
| AM16 | MG1655 Δ*xseA*Δ*uvrD* | | | This work | | |
| CT01 | MG1655 Δ*malK*::*kanR* | | | This work | | |
| CT02 | MG1655 *lexA3* | | | (99) | | |
| CT03 | MG1655 Δ*recN* | | | (32) | | |
| **Plasmid** | | | | | | **Source or Reference** |
| pUA66 | | Vector, SC101 ori, *kanR*, *gfpmut2* reporter | | | | (100) |
| pUA66 P_xseB_*-xseB* | | pUA66 P_xseB_*-xseB*, *kanR* | | | | This work |
| pUA66 P_xseA_*-xseA* | | pUA66 P_xseA_*-xseA*, *kanR* | | | | This work |
| pUA66 P_xseA_*-xseA*(F63A) | | pUA66 P_xseA_*-xseA*(F63A), *kanR* | | | | This work |
| pUA66 P_xseA_*-xseA*(D155A) | | pUA66 P_xseA_*-xseA*(D155A), *kanR* | | | | This work |
| pUA66 P_uvrD_*-uvrD* | | pUA66 P_uvrD_*-uvrD*, *kanR* | | | | This work |
| **Oligonucleotides to confirm genetic mutants and plasmids** | | | | | | |
| **Upstream Forward Primer (5'🡪3')** | | | **Downstream Reverse Primer (5'🡪3')** | | **Description** | |
| AGCATGGGCAATATCTCGAC | | | CTGCGGGGAATTTATTGAAA | | External primers for ∆*xseA* verification | |
| GCATGAGATGGGACAGGTTT | | | CCCGCTTTAACTTGCTTCAC | | Internal primers for ∆*xseA* verification | |
| GGCGCGGAAGTATACCACTA | | | GTCCAGCGTGTTTGTGCTAA | | External primers for ∆*xseB* verification | |
| AGCTTTGAAAAGGCGCTGAG | | | CCGGTGTAAAAGGGGTTAGA | | Internal primers for ∆*xseB* verification | |
| AAATGCCCGTGGTAACAGTG | | | ATATTGGGAACCGGCAAAAT | | External primers for ∆*exoX* verification | |
| AGGGAGGGATCGTTGAGATT | | | TTTCAGTGTTAAACGCAGCTC | | Internal primers for ∆*exoX* verification | |
| CCAGTTGCGACGTGGATATT | | | TTCCAGACGCTCTTTCTTGG | | External primers for ∆*recJ* verification | |
| AGTCGATGAAACGGCAGACT | | | TGGCCAGATATTGTCGATGA | | Internal primers for ∆*recJ* verification | |
| ATTGTGGCGCTAAAGCTGAT | | | AGCGGCGATGAGTTAGTGAT | | External primers for ∆*xonA* verification | |
| GTTAGATCGCCCTGCACAGT | | | CGATCTGCGTCACTGAAAAA | | Internal primers for ∆*xonA* verification | |
| GAAACCCTCAGCGAACTCAG | | | ACAACCACCAGAACGGCTAC | | External primers for ∆*sbcC* verification | |
| AAGAAAAAGCGCAACCTCAA | | | CAGTGCATAACCCGTCAATG | | Internal primers for ∆*sbcC* verification | |
| CCATGATTTGCCCTGTTGTA | | | GTGATACAGCGCCAGACAAA | | External primers for ∆*sbcD* verification | |
| TCACACCTCAGACTGGCATC | | | CTGAGTTCGCTGAGGGTTTC | | Internal primers for ∆*sbcD* verification | |
| CGTGGCGTTGATAAAGAACA | | | TCCAGGAACGTGGATTTACC | | External primers for ∆*recD* verification | |
| TAATGACAAACAGCGCGAAG | | | CAGACGCCCGTTATTGTTTT | | Internal primers for ∆*recD* verification | |
| ACGATCTCACCACTGACGTG | | | TACTGAAGATGGCGCAGATG | | External primers for ∆*uvrD* verification | |
| TAATGACAAACAGCGCGAAG | | | CGCCACCAGCCATTTAATAC | | Internal primers for ∆*uvrD* verification | |
| CCGTCTGGCTGCGGTAAATC | | | TGCCATCCTCACGGAACAGA | | Internal primers for ∆*malK*::*kanR* verification | |
| GCCAGGGGGTGGAGGATTTAAGC | | | CTATCTCCTGAGTCATTGCT | | External primers for ∆*malK*::*kanR* verification | |
| TTCCAAAATCGCCTTTTGCT | | | AGTGAGGAATGCCATGCAGA | | External primers for *lexA3* mutant verification | |
| ATGATGGATACTTTCTCGGCAGGAG | | | | | Internal primer of *kanR* used in conjunction of external primers to verify the deletion of genes | |
| **Oligonucleotides for plasmid construction (Gibson Assembly)** | | | | | | |
| **Plasmid/**  **Insert** | **Forward (5'🡪3')** | | **Reverse (5'🡪3')** | | **Description** | |
| P_xseB_*-xseB* | CCTTTCGTCTTCACCTCGAGCTTTGCTGTGCACATCACCT | | AGAGCTTGCATGCCTGCAGGTTACTCATTGTCCGGTGTAAAAG | | Amplify P_xseB_-*xseB* for integration into pUA66 using Gibson assembly | |
| pUA66 | CCTGCAGGCATGCAAGCTC | | CTCGAGGTGAAGACGAAAGGG | | Amplify pUA66 backbone to incorporate for Gibson Assembly with P_xseA_-*xseA* and P_xseB_-*xseB* | |
| P_xseA_-*xseA* | CCTTTCGTCTTCACCTCGAGGGGCAATATCTCGACCAG | | AGAGCTTGCATGCCTGCAGGTTAATGCACCTTTTTACGC | | Amplify P_xseA_-*xseA* for Gibson Assembly with pUA66 | |
| pUA66-P_xseA_-*xseA*(F63A) | CTGCGCGATGGCCCGCAACAGCA | | CGTACCTGGGCGGTGTCGTC | | Using pUA66-P_xseA_-*xseA* as a template, change F63A of *xseA* using Q5 site directed mutagenesis | |
| pUA66-P_xseA_-*xseA*(D155A) | GCGCTACATGCTATTTTGCATGTG | | AGCACCGGTTTTTGAGGT | | Using pUA66-P_xseA_-*xseA* as a template, change D155A of *xseA* using Q5 site directed mutagenesis | |
| P*_uvrD_*-*uvrD* | CCCTTTCGTCTTCACCTCGAGCGTTTTTACCGGCCTTTGGG | | CTGTCCATATGCACAGATGAGCGCATCAGCTACTCATTGG | | Amplify P_uvrD_-*uvrD* for integration into pUA66 using Gibson assembly | |
| **Oligonucleotides for strain construction via PCR and the Datsenko and Wanner Method** (95) | | | | | | |
| **PCR Fragment** | **Forward (5'🡪3')** | | **Reverse (5'🡪3')** | | **Description** | |
| FRT-*kanR*-FRT from pKD4 | TACAAAAAACCACTTCCCTCCCCTGCGCATTGCGTTGGTGTGATCACCTCAAAAACCGGTTAAGCTGGAGCTGCTTCGAAGTTCCTATACTTTCTAGAGAATAGGAACTTCGGAATAGGAACTTCCAAGAGACAGGATGAGGATCGTTTCGC | | TACTTCTGTCAGCACGGGCATGGCTTGATATCGAAAAAACGCGTTGAATTCGTGCTGGCTTAATTAGTTCCTATTCCGAAGTTCCTATTCTCTAGAAAGTATAGGAACTTCAGAGCGCTTTATTCTCACCAATAAAAAACGCCCGGCGGCAA | | Amplify FRT-*kanR*-FRT from pKD4 to transform into wild-type to create *xseA*_150_-FRT-*kanR*-FRT, which was subsequently cured using pCP20 | |
| *xseA* wild-type + mutation | TACAAAAAACCACTTCCCTCCCCTGCGCATTGCGTTGGTGTGATCACCTCAAAAACCGGT | | GAAACGATCCTCATCCTGTCTCTTGGAAGCAGCTCCAGCTTAATGCACCTTTTTACGCGATTTTTTTACT | | Primers to amplify *xseA* (wild-type and with mutations, except for F63A) from mutant plasmid for crossover PCR with *kanR* cassette, and ultimate transformation into *xseA*_150_ | |
| Kan from pUA66 | GCTGGAGCTGCTTCCAAGAGACAGGATGAGGATCGTTTC | | TACTTCTGTCAGCACGGGCATGGCTTGATATCGAAAAAACGCGTTGAATTCGTGCTGGCTTAAATTCTCACCAATAAAAAACGCCCGGCG | | Primers to amplify *kanR* from pUA66 for crossover PCR with *xseA* and ultimate integration into *xseA*_150_ | |
| *xseA*(F63A) | CTTGCTATCCCCGAAGGGCGGGTTACTATCGACTGAATAACCTGCTGATTTAGAATTTGATCTCGCTCACATGTTACCTTCTCAATCCCC | | GAAACGATCCTCATCCTGTCTCTTGGAAGCAGCTCCAGCTTAATGCACCTTTTTACGCGATTTTTTTACT | | Primers to amplify *xseA*-F63A from mutant plasmid for crossover PCR with *kanR* cassette, and ultimate transformation into ∆*xseA* cured strain | |
